# COVID-19 Vaccine Response in Allo-HSCT Recipients: Insights from a Real-World Prospective Cohort Study

**DOI:** 10.3390/vaccines13070726

**Published:** 2025-07-03

**Authors:** Emine Merve Savaş, Şeyma Yıldız, Zübeyde Nur Özkurt, Zehra Baltacı, Özlem Güzel Tunçcan, Zeynep Arzu Yeğin, Kayhan Çağlar, Nurdan Köktürk, Gonca Erbaş, Gülendam Bozdayı, Münci Yağcı

**Affiliations:** 1 Department of Hematology, Faculty of Medicine, Gazi University, Ankara 06560, Türkiye; seymayildiz@gazi.edu.tr (Ş.Y.); znozkurt@gazi.edu.tr (Z.N.Ö.); zbaltaci@gazi.edu.tr (Z.B.); zyegin@gazi.edu.tr (Z.A.Y.); ayagci@gazi.edu.tr (M.Y.); 2 Department of Infectious Disease and Clinical Microbiology, Faculty of Medicine, Gazi University, Ankara 06560, Türkiye; oguzel@gazi.edu.tr; 3 Department of Medical Microbiology, Faculty of Medicine, Gazi University, Ankara 06560, Türkiye; kcaglar@gazi.edu.tr (K.Ç.); gulendam@gazi.edu.tr (G.B.); 4 Department of Pulmonary Medicine, Faculty of Medicine, Gazi University, Ankara 06560, Türkiye; nkokturk@gazi.edu.tr; 5 Department of Radiology, Faculty of Medicine, Gazi University, Ankara 06560, Türkiye; goncaerbas@gazi.edu.tr

**Keywords:** Allo-HSCT, COVID-19 vaccination, antibody response, immunosuppression

## Abstract

**Background:** Allogeneic hematopoietic stem cell transplant (Allo-HSCT) recipients are still at increased risk of severe COVID-19 infection. Vaccination is a critical strategy to protect this population. This real-world prospective cohort study aimed to evaluate the immune response and clinical outcomes of COVID-19 vaccines in Allo-HSCT recipients. **Methods:** Allo-HSCT recipients (median age: 48 years) who received either the BNT162b2 or CoronaVac vaccines were included. Antibodies against the SARS-CoV-2 spike protein were quantitatively measured using the chemiluminescent microparticle immunoassay. Patient- and vaccine-related factors affecting antibody responses were analyzed. Adverse events, including graft-versus-host disease (GVHD) and post-vaccine infections, were recorded. **Results:** Among 95 Allo-HSCT recipients, 86.3% achieved adequate antibody responses following COVID-19 vaccination. Patients receiving ≥3 vaccine doses showed significantly higher antibody titers compared to those with only 2 doses (OR: 0.11; 95% CI: 0.02–0.53; *p* = 0.006 **). The use of Ruxolitinib or Ibrutinib was associate with increased odds of low antibody response (OR: 38.39; 95% CI: 3.14–468.95; *p* = 0.004 **). Hypogammaglobulinemia (low serum IgG levels) was associated with a reduced antibody response (OR: 0.17; 95% CI: 0.03–0.96; *p* = 0.045 *), while no significant correlation was found between serum IgA levels and antibody responses (*p* = 0.672). Three cases of post-vaccine GVHD were observed, and no fatalities related to COVID-19 occurred during the study. **Conclusions:** COVID-19 vaccination is safe and effective in Allo-HSCT recipients, with stronger responses especially following ≥3 vaccine doses. Patients receiving GVHD treatment or with hypogammaglobulinemia exhibited impaired responses, emphasizing the need for tailored vaccination strategies and close monitoring in this population.

## 1. Introduction

Allo-HSCT is a curative treatment method for various hematologic malignancies and disorders [1]. However, there is a significant increase in the risk of bacterial and viral infections due to various factors, including the use of immunosuppressive drugs during the post-transplant period and inadequate immune reconstitution [2,3]. Severe Acute Respiratory Syndrome Coronavirus 2 (SARS-CoV-2), the agent of the worldwide COVID-19 pandemic, remains a challenging issue for individuals undergoing Allo-HSCT. Even a mild infection can escalate rapidly into a life-threatening condition, necessitating hospitalization and potentially impacting overall survival [4,5,6]. Although the pandemic has been brought under control with COVID-19 vaccination, the immune response following vaccination may vary among Allo-HSCT recipients [7]. The lack of inclusion of individuals who have undergone Allo-HSCT and those with hematologic malignancies in vaccine trials resulted in an incomplete understanding of both the adverse effect profile and efficacy profile of the vaccines [8]. Due to the unique immunological challenges posed by the transplant procedure, transplant recipients may exhibit variable antibody responses following vaccination; factors such as the timing of vaccination post-transplant, the presence of graft-versus-host disease (GVHD), GVHD prophylaxis or treatments can all influence the efficacy of COVID-19 vaccines [9]. Consequently, it is important to adjust vaccination strategies for optimal protection against COVID-19, taking into account each patient’s unique medical history among Allo-HSCT recipients [10]. Given the limited data on COVID-19 vaccine response in Allo-HSCT recipients and the lack of clear identification of factors influencing antibody responses, this study aims to evaluate the antibody response and clinical implications of vaccination in this vulnerable population. By analyzing a real-world cohort, it helps address an important knowledge gap and inform future vaccination strategies in this high-risk group.

## 2. Material and Methods

### 2.1. Study Design and Setting

This prospective cohort study included patients with hematologic malignancies who underwent Allo-HCT between 2011 and 2021 at the Gazi University Bone Marrow Transplantation Unit and received at least two doses of a COVID-19 vaccine between January 2021 and April 2022. Antibody titers were measured at least 2 months after the final vaccine dose, due to the need to allow sufficient time for the development of an adequate immune response. Serum antibody levels were measured using a chemiluminescent microparticle immunoassay (CMIA) on the Abbott Architect i1000SR analyzer (Abbott Diagnostics, Chicago, IL, USA). Following the guidelines of the European Society for Blood and Marrow Transplantation (EBMT), routine COVID-19 vaccinations were administered to patients at least three months after transplantation [11].

### 2.2. Participants and Eligibility Criteria

Patients were included if they had received at least two doses of a COVID-19 vaccine. The exclusion criteria were prior COVID-19 infection (before or after transplantation), infection between vaccine doses or before antibody assessment, or receipt of only a single vaccine dose.

### 2.3. Vaccination Protocol

Vaccination was administered according to the national immunization program using CoronaVac (Sinovac Biotech, Beijing, China) or BNT162b2 (Pfizer–BioNTech, New York, NY, USA/Mainz, Germany). The first available vaccine in Turkey was CoronaVac. Thus, some patients received only CoronaVac, others received BNT after one dose of CoronaVac, and some were vaccinated with BNT alone. The subgroup analysis included those who received at least one dose of BNT. Additionally, a group of patients was vaccinated exclusively with BNT. For the subgroup analysis, patients who received at least one dose of BNT were included. Vaccination was postponed for patients with active GVHD, those receiving high-dose steroid therapy (≥0.5 mg/kg/day), or those with severe infections requiring hospitalization; as these patients were not vaccinated during the study period.

### 2.4. Data Sources and Variables

Transplant characteristics, current treatment information, and laboratory parameters including immunoglobulin levels were recorded to evaluate potential factors affecting antibody response.

### 2.5. Measurement of Antibody Response

Serum antibody levels were measured using a chemiluminescent microparticle immunoassay (CMIA) on the Abbott Architect i1000SR analyzer (Abbott Diagnostics, Chicago, IL, USA). This assay quantitatively detects IgG antibodies targeting the receptor-binding domain (RBD) of the SARS-CoV-2 spike (S1) protein. According to the manufacturer’s instructions, antibody concentrations ≥50 AU/mL were considered positive, and those <50 AU/mL were considered negative. However, based on prior studies in immunocompromised populations using the same assay [12,13], a more stringent cut-off of 300 AU/mL was adopted in this study to define an adequate antibody response. Values <300 AU/mL were classified as inadequate. The upper detection limit of the assay was 40,000 AU/mL. The upper limit of the vaccine response measured by the device was determined to be 40,000 IU/mL. Serum IgG and serum IgA concentrations were determined by the nephelometric method in accordance with the standard laboratory procedures.

### 2.6. Definitions

The Centre for International Blood and Marrow Transplant Research (CIBMTR) criteria determined the conditioning intensity [14]. The diagnosis and classification of acute graft-versus-host disease (aGvHD) were conducted by the 1994 Consensus Conference on Acute GvHD Grading [15], whereas the grading of chronic graft-versus-host disease (cGvHD) was performed following the NIH Consensus Criteria [16].

### 2.7. Statistical Analysis

Categorical variables were expressed as counts and percentages; continuous variables as medians and ranges. Normality was tested using Kolmogorov–Smirnov and Shapiro–Wilk tests. Depending on distribution, Student’s *t*-test, Mann–Whitney U, Kruskal–Wallis, or Chi-square tests were used. Multivariable logistic regression assessed associations between antibody response and covariates. Analyses were conducted using IBM SPSS v26.0; significance was defined as *p* < 0.05.

### 2.8. Endpoints

The primary endpoint was to identify predictors of antibody response post-vaccination. The secondary aim was to describe post-vaccine clinical events in Allo-HSCT recipients.

## 3. Results

### 3.1. Patient and Transplant Characteristics

A total of 95 patients were included in the study, comprising 61 males and 34 females, with a median age of 48 (range: 35–56) years. Among these patients, 49% had acute myeloid leukemia (AML), 20% had acute lymphoblastic leukemia (ALL), 7.4% had myelodysplastic syndrome (MDS), 7.4% had myeloproliferative neoplasia (MPN), and 12.4% underwent transplantation for various other reasons (such as MM and CLL). Of the total, 66 patients (69.5%) received transplants from fully matched donors. Myeloablative conditioning regimens were administered to 50.5% (48 patients), while 49.5% (47 patients) underwent non-myeloablative or reduced-intensity conditioning (RIC) regimens. For GVHD prophylaxis, 60% (57 patients) received cyclosporine and methotrexate (CSA-MTX), 40% (38 patients) received cyclosporine and mycophenolate mofetil (CSA-MMF), and in addition, 11 patients (11.6%) were treated with anti-thymocyte globuline (ATG), while 13 patients (13.7%) received post-transplant cyclophosphamide (PTCy). Detailed patient and transplant characteristics can be found in Table 1.

### 3.2. Vaccination and Serologic Responses

Out of the patients, 27 (28.4%) received CoronaVac, while 40 received BNT162b2, and 28 patients received CoronaVac initially and then BNT162B2. A total of 37 patients received two doses of vaccine, 34 received three doses, and 24 received four or five doses. The time interval from transplantation to the first vaccine dose was 1269 days (range: 650–2487), and the time interval from the last vaccine to the time of antibody measurement was 128 days (72–211). The median antibody titer was 13,367 (760–40,000). Thirteen patients (13.6%) were classified as low or non-responders. Among these, 6 were on Ruxolitinib, 2 on Ibrutinib, 1 on cyclosporine, and 4 were not receiving any immunosuppressive therapy.

#### 3.2.1. Vaccination Number

**Figure 1:**
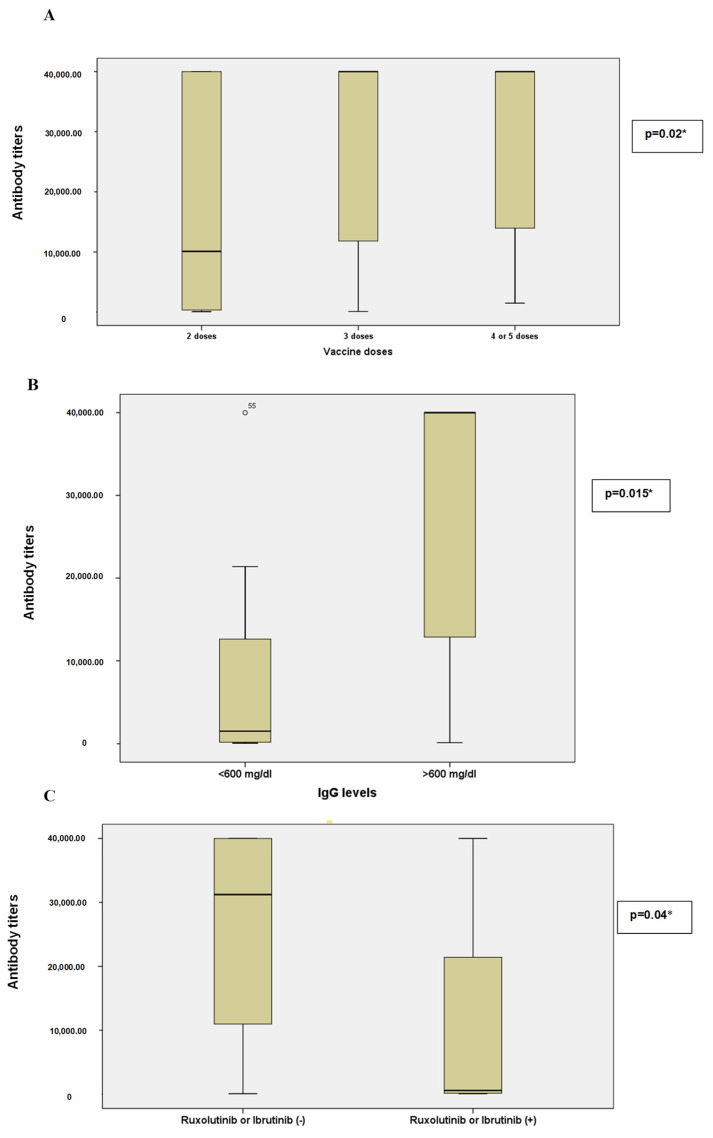
A comparison of post-vaccination SARS-CoV-2 antibody titers based on clinical variables. (**A**) Antibody titers stratified by the number of COVID-19 vaccine doses received (2, 3, or ≥4 doses). (**B**) Antibody titers according to serum immunoglobulin G (IgG) levels (<600 mg/dL vs. ≥600 mg/dL). (**C**) Antibody titers in patients receiving versus not receiving Ruxolitinib or Ibrutinib. Horizontal lines represent medians; box plots indicate interquartile ranges (IQR), and whiskers denote minimum and maximum values excluding outliers. Statistical comparisons were performed using the Kruskal–Wallis test for panel A and Mann–Whitney U test for panels B and C. Antibody levels were measured in arbitrary units per milliliter (AU/mL), with a measurement range from 0 to 40,000 AU/mL.

Median antibody titers increased with the number of vaccine doses: 10,094 AU/mL (345.6–40,000) for 2 doses, 40,000 AU/mL (11,818–40,000) for 3 doses, and 40,000 AU/mL (13,965–40,000) for 4–5 doses. Booster vaccinations were associated with significantly higher antibody responses (*p* = 0.02 *, see Figure 1A).

#### 3.2.2. Vaccination Time

Eleven patients received vaccination within the first 6 months post-transplantation, six patients between 6 and 12 months, and seventy-seven patients one year or more after transplantation. Median antibody titers were 302 AU/mL (126–12,039) for 3–6 months, 23,067 AU/mL (2319–40,000) for 6–12 months, and 13,965 AU/mL (1359–40,000) for >12 months. Antibody levels in patients vaccinated within 3–6 months were lower, with borderline statistical significance (*p* = 0.065).

#### 3.2.3. Vaccination Type

Patients who received at least one dose of BNT162b2 had significantly higher antibody levels compared to those who received only CoronaVac [23,555 AU/mL (5457–40,000) vs. 827 AU/mL (448–7516), *p* < 0.001 ***]. Due to the markedly lower titers in the CoronaVac-only group, subgroup analyses were performed among recipients of at least one dose of BNT162b2.

### 3.3. Predictors of Antibody Response

Lower antibody responses were observed in individuals with low serum IgA levels compared to those with normal serum IgA levels [1895 (147–31,696) vs. 37,604 (10,232–40,000), *p* = 0.006 **]. Similarly, individuals with low serum IgG levels exhibited lower antibody titers [682 (192.5–12,202) vs. 20,519 (2260–40,000), *p* < 0.015 *; see Figure 1B].

Patients receiving immunosuppressive therapy for acute or chronic GVHD or for maintenance were analyzed. A total of 11 patients were under treatment for chronic GVHD: 8 with Ruxolitinib, 2 with Ibrutinib, and 1 with both. Additionally, 11 patients received maintenance therapy (5 on TKIs, 4 on FLT-3 inhibitors, 1 on a BCL-2 inhibitor, and 1 on a proteasome inhibitor).

Patients treated with Ruxolitinib or Ibrutinib had significantly lower antibody titers than those not receiving these agents [296 (117–23,815) vs. 31,220 (10,510–40,000), *p* = 0.004 **, see Figure 1C]. When analyzed separately, patients receiving only Ruxolitinib [126 (108–21,479), *p* = 0.046 *] or only Ibrutinib [296 (229–891), *p* = 0.015 *] had significantly lower titers than non-users. No significant differences were found between patients not receiving maintenance treatment and those treated with TKIs or FLT-3 inhibitors [31,220 (8583–40,000), 1895 (589–12,875), and 28,300 (11,855–37,604), respectively; *p* = 0.23].

No significant difference in antibody responses was observed between patients who received DLI in the past 2 years and those who did not [6410 (982–25,463) vs. 27,488 (16,356–40,000), *p* = 0.14]. The use of ATG [31,089 (11,818–40,000) vs. 23,484 (4161–40,000), *p* = 0.62] or PTCy [29,300 (687–40,000) vs. 23,555 (7631–40,000), *p* = 0.76] did not significantly affect antibody titers.

Variables that showed statistically significant associations in univariate analysis—including serum IgA and IgG levels, the use of Ruxolitinib or Ibrutinib, and the number of vaccine doses—were selected for multivariable logistic regression to identify independent predictors of antibody response. Antibody titers were dichotomized at the median for this analysis. Receiving three or more vaccine doses was independently associated with higher antibody responses (OR: 0.11, 95% CI: 0.02–0.53, *p* = 0.006 **). Conversely, treatment with Ruxolitinib or Ibrutinib was strongly associated with lower antibody titers (OR: 38.39, 95% CI: 3.14–468.95, *p* = 0.004 **). Similarly, low serum IgG levels were significantly associated with reduced antibody responses (OR: 0.17, 95% CI: 0.03–0.96, *p* = 0.045 *), whereas serum IgA levels were not significantly associated (OR: 0.66, 95% CI: 0.10–4.58, *p* = 0.672). As shown in Table 2, these results indicate that receiving a booster vaccination with three or more doses is an independent positive predictor of antibody response, while treatment with Ruxolitinib or Ibrutinib and low serum IgG levels are independent negative predictors.

### 3.4. COVID Infections and Safety Outcomes

A total of 21 patients (22.3%) experienced a COVID-19 infection following vaccination. Among them, seven patients had received CoronaVac (33.33%), eight had received BNT162b2 (38.10%), and six had initially been vaccinated with CoronaVac followed by BNT162b2 (28.57%). Notably, one patient did not exhibit any post-vaccination antibody response, while the remaining 20 patients showed a median post-vaccination antibody response of 1487 (range: 187–16,384). Among these, five patients required hospitalization, with two of them being admitted to the intensive care unit. None of the patients who were vaccinated against COVID-19 have died due to infection from the virus in this cohort.

Acute GVHD developed in three patients after COVID-19 vaccination (two following BNT162b2 and one following CoronaVac and subsequent BNT162b2 vaccination). Among these patients, one had a history of COVID-19 infection post-vaccination, which was followed by acute cutaneous GVHD. Subsequently, this patient was diagnosed with bronchiolitis obliterans (BO) and died because of rapidly progressing right heart failure, which was related to BO. The other two patients were diagnosed with cutaneous and hepatic GVHD, and hepatic GVHD, respectively, and were managed with short-term steroid treatment. During a three-month follow-up post-COVID vaccination, there were no reported cases of relapse associated with the vaccination.

## 4. Discussion

In this prospective real-life cohort study, we evaluated the antibody response to COVID-19 vaccination in 95 patients who underwent Allo-HSCT. One of the most prominent findings was the achievement of adequate vaccine-induced antibody responses in a high proportion of patients (86.3%), particularly after the initial six months post-vaccination. These findings are consistent with previously reported outcomes in similar cohorts of Allo-HSCT recipients [13,17].

Importantly, our findings demonstrate that antibody responses are significantly enhanced with booster vaccination (2 doses vs. ≥3 doses). This observation is consistent with prior findings demonstrating that a third vaccine dose enhances vaccine-induced immune responses while maintaining an acceptable safety profile in immunocompromised populations [18,19,20]. In a recent study with Allo-HCT recipients, booster vaccination similarly improved both humoral and cellular immune responses [21]. Therefore, administering a third dose of the vaccine may enhance the antibody response in patients in the early post-transplant period, during GVHD prophylaxis, or in individuals receiving treatment for chronic GVHD.

Regarding the timing of vaccination, patients vaccinated within 3–6 months after transplantation showed slightly lower antibody responses compared to those vaccinated later, although the difference did not reach statistical significance. This finding suggests a potential reduction in vaccine responsiveness when vaccination is administered early after transplantation. Current immunization guidelines recommend initiating vaccination preferably after 6 months post-transplant when feasible, to ensure optimal immune reconstitution [11,22]. Nevertheless, evidence from a large multicenter trial supports the efficacy of early vaccination, even within the first month, demonstrating adequate antibody responses in this setting [23]. Our data partially support these findings, while also highlighting the potential benefits of postponing vaccination to ≥6 months when clinically appropriate.

We also investigated factors affecting humoral vaccine responses. Although there was heterogeneity in transplant timelines, the use of tyrosine kinase inhibitors (TKIs), anti-thymocyte globulin (ATG), or post-transplant cyclophosphamide (PTCY) was not associated with impaired antibody responses. In contrast, patients who were actively receiving or had received Ruxolitinib or Ibrutinib within the past 6 months showed a marked reduction in antibody levels, which was a strong independent negative factor for the antibody response to vaccination (OR: 38.39, 95% CI: 3.14–468.95, *p* = 0.004 **). This finding is clinically relevant, as an inadequate vaccine-induced antibody response may lead to adverse clinical outcomes in patients receiving immunosuppressive treatment for GVHD.

The known immunological effects of JAK and BTK inhibitors may explain these findings. JAK inhibitors impair B cell maturation, reduce antibody production, inhibit T cell proliferation, and weaken NK cell function—collectively leading to reduced immune competence [24]. Similarly, BTK inhibitors primarily target B cells by disrupting B cell receptor signaling, leading to impaired activation, differentiation, and antibody production [25]. Several studies have demonstrated that both JAK inhibitors and BTK inhibitors impair vaccine-induced antibody responses. In a meta-analysis including 11,086 patients with hematological malignancies evaluating SARS-CoV-2 vaccine antibody responses, significantly impaired responses were observed in individuals receiving Ruxolitinib or BTK inhibitors [26]. In a series of 70 patients who underwent Allo-HSCT due to AML, it was similarly observed that individuals using Ibrutinib or Ruxolitinib did not generate vaccine antibody responses [13]. In another study investigating COVID-19 vaccine antibody responses in patients with chronic lymphocytic leukemia, individuals receiving BTK inhibitors showed markedly reduced humoral and cellular immune responses [27]. These findings highlight the importance of individualized vaccine strategies in Allo-HSCT recipients, especially in those receiving post-transplant immunosuppressive therapy, as their vaccine responses may be compromised not only to COVID-19 vaccines but also to other essential post-transplant vaccines. Currently, there are no clear recommendations in the literature regarding this issue; however, based on our findings, booster vaccination may offer a potential solution. Nonetheless, further studies are needed to validate this approach.

Hypogammaglobulinemia is a frequent immunologic complication after Allo-HSCT, often resulting from impaired B-cell reconstitution, chronic graft-versus-host disease, or prolonged immunosuppressive therapy. It reflects a fundamental defect in humoral immunity and is associated with increased susceptibility to infections [28]. In our study, patients with hypogammaglobulinemia showed significantly lower vaccine-induced IgG responses, which is expected given the reduced baseline capacity for immunoglobulin synthesis. Since COVID-19 vaccines primarily induce IgG-class antibodies, this impairment directly affects serologic response. Similar findings have been reported in patients with chronic lymphocytic leukemia and other B-cell malignancies, where hypogammaglobulinemia has been consistently associated with poor vaccine immunogenicity [29,30]. Therefore, evaluating serum IgG levels may serve as a practical marker for identifying individuals who may benefit from modified vaccination strategies, such as additional booster doses or adjunctive prophylaxis.

The development of graft-versus-host disease (GVHD) was observed in three patients following COVID-19 vaccination. In one patient, the occurrence of GVHD shortly after COVID vaccination, and subsequently, a COVID infection, led to the consideration that the clinical manifestation of GVHD might be associated with the primary infection. The incidence of GVHD in this patient group was deemed acceptable and comparable to rates reported in other studies [23].

The limitations of this study include the use of different types of vaccines, variations in the duration since transplant for patients, and differences in the timing of antibody response assessments following vaccination. Additionally, the relatively small sample size of the group using immunosuppressive agents suggests that these results should be strengthened and validated through large-scale studies.

In conclusion, based on the results of the real-life cohort of 95 individuals; post-COVID vaccination antibody development can be observed in the majority of Allo-HSCT recipients. However, vaccine antibody responses are diminished in individuals with chronic graft-versus-host disease (GVHD), especially those using Ruxolitinib or Ibrutinib, or experiencing hypogammaglobulinemia. Booster vaccinations may be crucial, particularly in these groups. Another important finding of our study is that COVID-19 vaccination appears to be safe in Allo-HSCT recipients, particularly regarding the risk of GVHD. These findings should be validated in larger patient cohorts, and additional studies assessing antibody responses to other vaccines should also be conducted.

## Figures and Tables

**Table 1:** Patient and transplant characteristics (n = 95).

**Age (years) [median (range)]**	48 (35–56)
**Gender (male/female) [n (%)]**	61 (64.2)/34(35.8)
**Diagnosis [n (%)]**	
AML	49 (51.6)
ALL	20 (21.1)
MDS	7 (7.4)
MPN	7 (7.4)
Others	12 (12.5)
**Pretransplant Disease Status [n (%)]**	
Complete remission	74 (77.9)
Partial remission	2 (2.1)
Stable disease	7 (7.4)
Progressive disease	12 (12.6)
**Donor Type [n (%)]**	
MRD	66 (69.5)
MMRD	1 (1.1)
MUD	8 (8.4)
MMUD	12 (12.6)
Haploidentical	8 (8.4)
**Conditioning Regimen [n (%)]**	
Myeloablative	48 (50.5)
Nonmyeloablative-RIC	47 (49.5)
**GVHD Prophylaxis I [n (%)]**	
CSA-MTX	57 (60)
CSA-MMF	38 (40)
**GVHD Prophylaxis II [n (%)]**	
ATG-based	11 (11.6)
PtCy-based	13 (13.7)
**Acute GVHD [n (%)]**	8 (8.4)
**Chronic GVHD [n (%)]**	15 (15.8)
**Vaccination Type [n (%)]**	
CoronaVac	27 (28.4)
BNT162b2	40 (42.1)
CoronaVac + BNT162b2	28 (29.5)
**Vaccination Number [n (%)]**	
2 doses	37 (38.9)
3 doses	34 (35.8)
4 or 5 doses	24 (25.3)
**Transplant to first vaccination time (days) [median (range)]**	1269 (650–2487)
**First–last vaccination time (days) [median (range)]**	
2 vaccinations	28 (28–132)
3 vaccinations	133 (121–150)
4 or 5 vaccinations	265 (251–296)
**Antibody screening–last vaccination time (days) [median (range)]**	128 (72–211)

n (%) indicates the number of patients and corresponding percentage. Age is presented as median (range). Abbreviations: AML—acute myeloid leukemia; ALL—acute lymphoblastic leukemia; MDS—myelodysplastic syndrome; MPN—myeloproliferative disease; MRD—Matched Related Donor; MMRD—Mismatched Related Donor; MUD—Matched Unrelated Donor; MMUD—Mismatched Unrelated Donor; RIC—Reduced Intensity Regimen.

**Table 2:** Results of multivariable logistic regression analysis *.

Variable	OR (Exp(B))	95% CI (Lower-Upper)	*p*-Value
Jakavi or Ibrutinib use	38.39	3.14–468.95	0.004 **
Vaccine doses (2 doses OR ≤3 doses)	0.11	0.02–0.53	0.006 **
Serum IgG level (low/high)	0.17	0.03–0.96	0.045 *
Serum IgA level (low/high)	0.66	0.10–4.58	0.672

Odds ratios (OR) and 95% confidence intervals (CI) are presented. Statistically significant at *p* < 0.05 (*), highly significant at *p* < 0.01 (**).

## Data Availability

Data are contained within the article.
